# A novel noninvasive prenatal testing method for chromosomal rearrangements using maternal circulating cell‐free foetal DNA

**DOI:** 10.1002/ctm2.1160

**Published:** 2023-01-07

**Authors:** Shuo Zhang, Zhenle Pei, Caixia Lei, Yixuan Bai, Junping Wu, Min Xiao, Jing Zhou, Bin Hu, Songchang Chen, Yiming Wu, Jingmin Yang, Xiaoxi Sun, Daru Lu, Chenming Xu, Congjian Xu

**Affiliations:** ^1^ Shanghai Ji Ai Genetics & IVF Institute Shanghai Key Laboratory of Female Reproductive Endocrine Related Diseases Obstetrics and Gynecology Hospital of Fudan University Shanghai China; ^2^ State Key Laboratory of Genetic Engineering School of Life Science Fudan University Shanghai China; ^3^ NHC Key Laboratory of Birth Defects and Reproductive Health Chongqing Key Laboratory of Birth Defects and Reproductive Health Chongqing Population and Family Planning Science and Technology Research Institute Chongqing China; ^4^ Department of Obstetrics and Gynecology of Shanghai Medical School Fudan University Shanghai China

Dear Editor,

At present, there is no noninvasive prenatal testing (NIPT) method available for pregnant women that one partner of couples carry balanced chromosomal rearrangements (BCRs). Here we present a novel NIPT method for detecting foetal chromosomal rearrangements based on haplotype analysis of maternal cell‐free foetal DNA (cffDNA) and demonstrate its effectiveness and potential for clinical application in a cohort of 46 patients.

BCRs are estimated to occur in approximately 0.5% of newborn infants^1^ and the prevalence is even up to 4%–5% in individuals with fertility problems.^2^ BCRs can result in infertility, recurrent miscarriages, or offspring with congenital malformations.^3^, ^4^ In general, invasive prenatal diagnosis (IPD) such as amniocentesis is recommended after pregnancy for couples in which one partner carries BCRs. However, IPD poses a 0.5% risk of miscarriage, infection and preterm labour.^5^ Therefore, many couples decline IPD despite being BCR carriers. During pregnancy, maternal plasma contains a small percentage of cffDNA, which is an easily accessible source for the noninvasive determination of foetal chromosomal anomalies. The discovery has presented the possibility of noninvasive testing for genetic diseases; NIPT is now being increasingly used for detecting foetal chromosomal aneuploidies and monogenic disorders.^6^, ^7^ However, NIPT for structural chromosomal rearrangements (NIPT‐SR) has not been available until now, despite its relatively high incidence.

Core family‐based haplotype linkage analysis has long been used to indirectly diagnose monogenic diseases, such as in preimplantation genetic testing (PGT) and NIPT.^7^, ^8^ In recent years, our team has illustrated haplotype analysis can also accurately detect balanced and unbalanced chromosome structural rearrangements in human embryos through PGT.^9^ The concept that foetal karyotypes can be predicted by inherited parental haplotypes was first introduced for NIPT in this study. The general workflow of our study was illustrated in Figure 1A. A total of 46 patients were recruited and participated in this research finally, peripheral blood of the couple and one patient's relative was collected, the karyotyping results of patients (Table 1) and their parents (Table S1) are shown. To achieve a highly accurate haplotype, we designed a capture‐based single nucleotide polymorphism (SNP) panel that spanned 5.1 Mb regions and included 65 172 SNPs across the genome, with a median distance of 40 kb between each neighbouring SNP. Minor allele frequencies of these SNPs were more than 0.3 and the VIF threshold was set to 1.5, which could generate more informative markers and facilitate identification of the crossover more accurately. By library preparation and massively parallel sequencing, the average sequencing error rate was approximately 0.11%, indicating the high fidelity of data quality (Figure S1).

**FIGURE 1:**
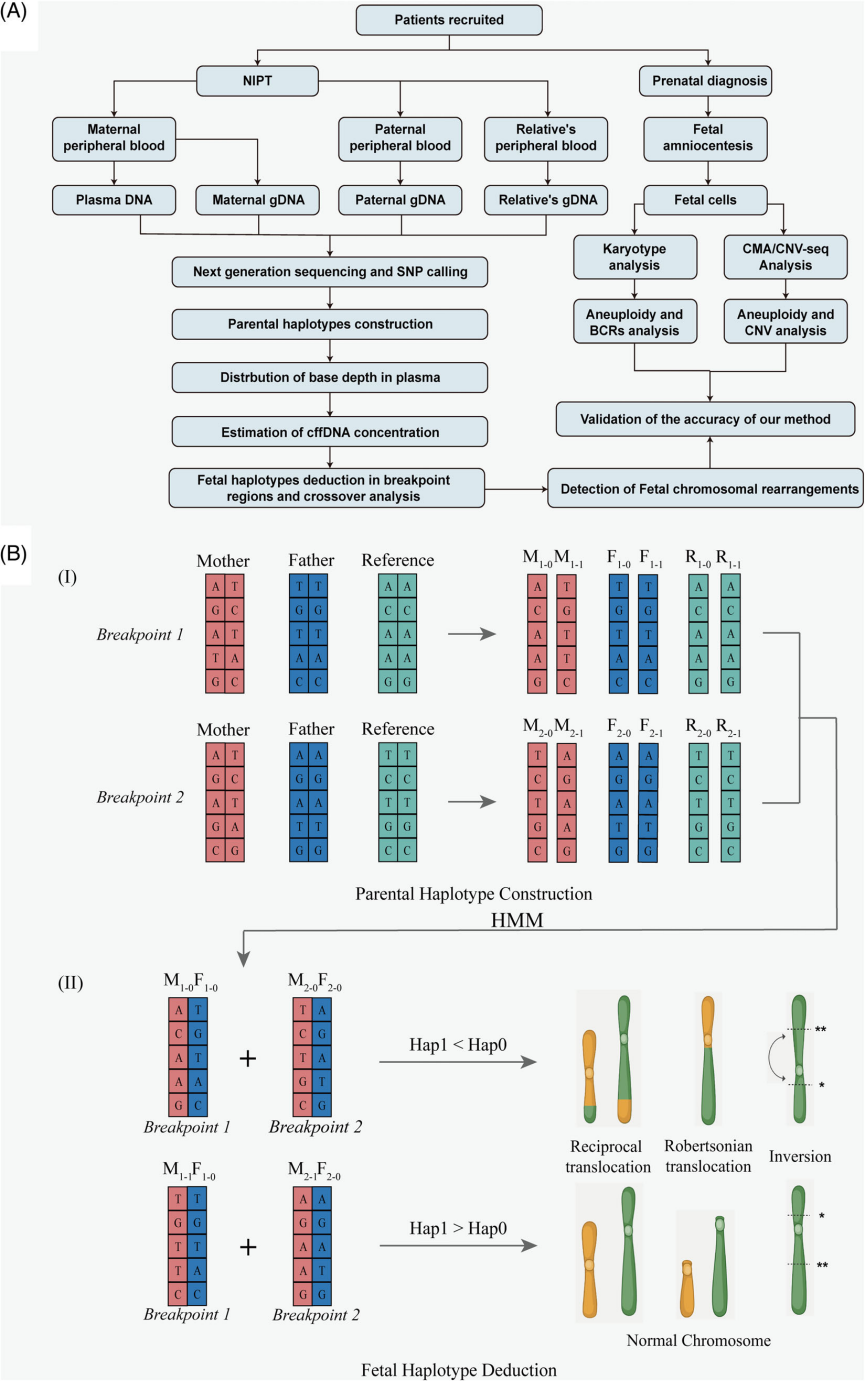
The workflow of this study and the process of haplotype construction and deduction. (A) The designation and workflow of this study. First, we collected peripheral blood samples from patient couples and one relative. The genotypes of both parents and the reference were determined by capture enrichment and massive parallel sequencing. Then, noninvasive screening for and common aneuploidies and BCRs, amniocentesis prenatal diagnosis was blindly conducted by two independent groups. Finally, the accuracy of this haplotype‐based noninvasive prenatal testing method was evaluated by invasive amniocentesis prenatal diagnosis. (B) The process of haplotype construction and deduction, the genotypes of both parents and the reference were determined by capture enrichment and massive parallel sequencing. I: The process of parental haplotype construction. Parental haplotypes were constructed using sequencing data from the parents and a reference, either one grandparent or a sibling could be used as the reference. II: The process of foetal haplotype deduction. Using the parental haplotypes, foetal haplotypes were inferred with a hidden Markov model (HMM) through maternal plasma DNA sequencing data. Finally, the foetal karyotypes were predicted by the inherited haplotypes that covered the rearrangements breakpoint regions. Hap1 represents the haplotypes with normal karyotype; Hap0 represents the haplotypes with rearranged karyotype. M: mother; F: father; R: reference; HMM, hidden Markov model; Hap: haplotype

**TABLE 1:** The detailed characteristics and NIPT results of patients enrolled in this study

Patient	Gestational age (weeks)	Maternal age/Paternal age	Karyotypes of patients by peripheral blood analysis^a^	Indications for karyotyping	Foetal fraction of cfDNA (%)	NIPT results of foetal karyotype	Prenatal diagnosis or PGT results
**Reciprocal translocation**							
Patient‐1	13	26/28	46,XX,t(1;3)(q31;q23),mat	Recurrent miscarriage	16.5	Normal	Normal
Patient‐2	12	33/36	46,XX,t(1;5)(q32;q33),pat	Infertility	14.8	Normal	Normal
Patient‐3	16	27/27	46,XY,t(1;10)(q21;q26),pat	Infertility	10.1	BCR carrier	BCR carrier
Patient‐4	22	31/31	46,XY,t(1;12)(q21;p11),pat	Recurrent miscarriage	23.4	BCR carrier	BCR carrier
Patient‐5	19	31/35	46,XX,t(1;17)(p22;q23),pat	Infertility	15.6	Normal	Normal
Patient‐6	18	38/43	46,XX,t(1;20)(q25;p11.2),mat	One miscarriage	18.2	Normal	Normal
Patient‐7	23	33/35	46,XX,t(2;6)(q31;p25),mat	Recurrent miscarriage	23.4	Normal	Normal
Patient‐8	21	25/27	46,XX,t(2;8)(q33;q13), mat	Recurrent miscarriage	21.1	Normal	Normal
Patient‐9	23	24/24	46,XY,t(2;10)(q35;q22.1), mat	Infertility	25.9	Normal	Normal
Patient‐10	13	32/35	46,XX,t(2;10)(p21;p15), mat	One miscarriage	10.5	Normal	Normal
Patient‐11	18	35/34	46,XY,t(3;4)(p21;q35), mat	One miscarriage	19.7	BCR carrier	BCR carrier
Patient‐12	18	35/29	46,XY,t(3;7)(p11;q36),mat	Recurrent miscarriage	13.8	BCR carrier	BCR carrier
Patient‐13	12	28/31	46,XY,t(3;7)(p21;p15), pat	Recurrent miscarriage	10.7	Normal	Normal
Patient‐14	26	26/42	46,XX,t(3;6)(p10;p10),mat	One miscarriage	26.7	Normal	Normal
Patient‐15	20	36/37	46,XX,t(4;16)(q25;q13), pat	Infertility	26.0	Normal	Normal
Patient‐16	17	32/32	46,XX,t(4;18)(p14;p11), mat	Affected foetus	15.4	Normal	Normal
Patient‐17	13	29/30	46,XY,t(5,18)(q15,p11.2),mat	Infertility	4.6	BCR carrier	BCR carrier
Patient‐18	12	30/32	46,XX,t(5;13)(q15;p12), pat	Infertility	9.3	BCR carrier	BCR carrier
Patient‐19	28	29/30	46,XY,t(6;10)(q24;q23),mat	Recurrent miscarriage	36.7	Normal	Normal
Patient‐20	13	30/31	46,XY,t(9;12)(q31;q14),mat	Affected foetus	13.7	Normal	Normal
Patient‐21	25	34/37	46,XY,t(9;15)(p23;q25),mat	Recurrent miscarriage	20.1	Normal	Normal
Patient‐22	13	28/31	46,XX,t(9;17)(q34.2;p11.2),mat	Recurrent miscarriage	16.7	BCR carrier	BCR carrier
Patient‐23	14	27/28	46,XY,t(9,22)(q22,q11.2), pat	Recurrent miscarriage	11.9	Normal	Normal
Patient‐24	20	32/33	46,XX,t(10;17)(q26.3;q24), mat	Affected foetus	18.1	Normal	Normal
Patient‐25	18	27/31	46,XY,t(12;21)(p11.1;p11.1),pat	Recurrent miscarriage	17.5	BCR carrier	BCR carrier
Patient‐26	19	31/34	46,XX,t(13;14)(q32;q24),mat	One miscarriage	22.3	Normal	Normal
Patient‐27	15	35/36	46,XX,t(13;21)(q22;q22.1), mat	One miscarriage	12.9	Normal	Normal
Patient‐28	15	29/32	46,XX,t(13;22)(q21.2;q13.1), mat	Recurrent miscarriage	10.3	Normal	Normal
Patient‐29	13	30/33	46,XY,t(7;11)(q21.3;q21), unknown	Infertility	13.9	BCR carrier	BCR carrier
Patient‐30	12	34/35	46,XX,t(2;11) (q31;p14), unknown	Infertility	7.1	Normal	Normal
**Robertsonian translocation**							
Patient‐31	25	32/31	45,XX,rob(13;14)(q10;q10), pat	Recurrent miscarriage	30.9	Normal	Normal
Patient‐32	14	38/41	45,XX,rob(13;14)(q10;q10), mat	One miscarriage	9.6	BCR carrier	BCR carrier
Patient‐33	15	36/35	45,XX,rob(13;14)(q10;q10),mat	Recurrent miscarriage	8.8	BCR carrier	BCR carrier
Patient‐34	24	27/30	45,XY,rob(13;14)(q10;q10), mat	One miscarriage	27.4	Normal	Normal
Patient‐35	12	28/30	45,XX,rob(13;14)(q10;q10),mat	Infertility	12.3	Normal	Normal
Patient‐36	23	34/35	45,XY,rob(13;14)(q10;q10),pat	Infertility	20.7	BCR carrier	BCR carrier
Patient‐37	15	29/31	45,XX,rob(13;14)(q10;q10),pat	Recurrent miscarriage	7.9	Normal	Normal
Patient‐38	23	32/33	45,XY,rob(14;15)(q10;q10),pat	One miscarriage	14.9	BCR carrier	BCR carrier
Patient‐39	24	32/30	45,XX,rob(14;21)(q10;q10), pat	Recurrent miscarriage	21.1	Normal	Normal
Patient‐40	11	31/35	45,XX,rob(14;21)(q10;q10),pat	Recurrent miscarriage	5.7	Normal	Normal
Patient‐41	19	32/32	45,XY,rob(14;21)(q10;q10),pat	Infertility	21.2	Normal	Normal
Patient‐42	19	31/31	45,XY,rob(14;21)(q10;q10),mat	Recurrent miscarriage	17.1	Normal	Normal
Patient‐43	23	34/33	45,XY,rob(14;21)(q10;q10),pat	Recurrent miscarriage	16.2	BCR carrier	BCR carrier
**Chromosomal inversion**							
Patient‐44	13	32/32	46,XY,inv(7)(p21q21),pat	Recurrent miscarriage	20.8	Normal	Normal
Patient‐45	17	33/33	46,XX,inv(8)(p22q22), pat	Recurrent miscarriage	19.2	Normal	Normal
Patient‐46	12	29/31	46,XY,inv(18)(p11.2q21.1), mat	Recurrent miscarriage	5.6	Normal	Normal

^a^ In patients 29 and 30, the karyotypes of patient's parents were unknown, their children were used as reference to phase haplotype. Patients 22 and 29 were naturally pregnant.

To determine the parental allele inherited by the foetus, we first established the parental haplotypes through target‐region sequencing genotyping data of the parents and one relative based on Mendel's law (Figure 1B). This strategy successfully helped construct the haplotypes on each chromosome carrying structural variants among the family members. After constructing the phased parental haplotypes, the foetal inheritance status could be inferred based on hidden Markov model (Figure 1B). All foetal haplotypes were successfully inferred in our cases, foetal karyotypes were then predicted according to the haplotypes transmitted around the rearrangement breakpoint regions. For example, in patient 6 (Figure 2), the pregnant woman was a balanced translation carrier and inherited the aberration from her mother (Figure 2B and C), targeted sequencing was conducted for the couple and grandmother and SNPs were then used to construct the parental haplotype (Figure 2D). For the inferred fatal haplotypes that covered the two suggested breakpoints, we found that the foetus inherited both the maternal haplotype M1, which was linked to the normal chromosome (Figure 2E and F). Thus, the foetus was predicted to have a normal karyotype, not be a BCR carrier (Figure S2). In addition, we identified one crossover in chromosome 1 and two crossovers in chromosome 20, the positions of the crossovers were accurately identified compared with the standard haplotype using the foetal genomic DNA.

**FIGURE 2:**
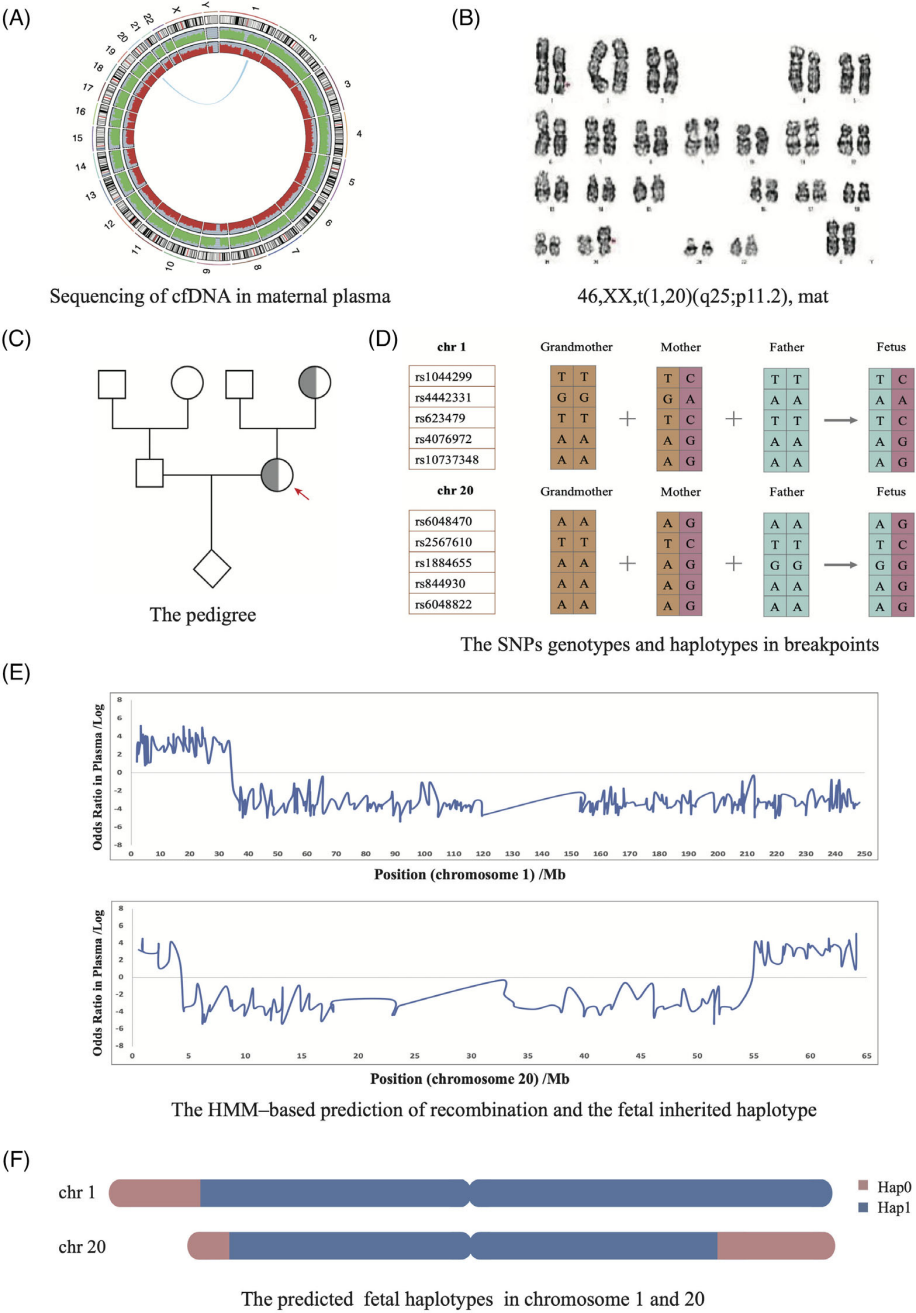
The noninvasive testing results of patient 6. (A) The circle plot of sequencing date of total cell‐free DNA in maternal plasma. (B) The karyotype of this pregnant woman was t(1;20)(q25;p11.2) and inherited from her mother. (C) The pedigree of this enrolled family. (D) The informative SNPs used to construct the parental haplotype of the two rearrangements breakpoint regions. Targeted sequencing was conducted in the couple and grandmother, the SNPs that were heterozygous in the mother and homozygous in the father and grandmother were defined as informative SNPs. (E) Identification of recombination breakpoints and haplotype assembly. The *x*‐axis represents the position on chromosome 1 and 20, the *y*‐axis represents the logarithmic values of the odds ratios of foetal inheritance of the maternal haplotypes. The entire genomic region of chromosome 1 and 20 are shown with lines indicating the logarithmic odds ratio between transmission probability of ‘Hap1’ alleles and ‘Hap0’ alleles. When an odds ratio is greater than 0, the haplotype is defined as ‘Hap0’, whereas when a value is less than 0, the haplotype is defined as ‘Hap1’. (F) The predicted foetal haplotype in chromosome 1 and 20. ‘Hap 0’ alleles are linked to the rearranged chromosome, while ‘Hap1’ alleles are linked to the normal chromosome. Therefore, the foetus was thus predicted to be with a normal karyotype, not a carrier of t(1,20)(q25;p11.2). HMM, hidden Markov model; Hap: haplotype

To further estimate the accuracy of the inferred foetal haplotype using this NIPT method, SNP genotypes those inferred from maternal plasma DNA sequencing were validated using direct sequencing results of foetal genomic DNA obtained from amniocentesis or blood. The average accuracy of inferred maternal and paternal alleles was 98.8% (Table S2), which was consistent with that reported in previous publications.^10^ Overall, an average of 1.63 informative SNPs were obtained in each Mb distance, and the positions of crossovers in the foetus could be accurately identified within one Mb. The distribution of total SNPs, average inferred SNPs and informative SNPs in each chromosome was shown (Figure S3), respectively.

To validate the effectiveness of this proposed NIPT method, the results were confirmed by karyotyping analysis of foetal cells. Among all patients, 29 pregnant women underwent invasive prenatal amniocentesis in the second trimester and 7 pregnant women had their newborns' umbilical cord blood testing at birth, our results revealed that all foetal karyotypes were completely consistent with those detected using the NIPT method (Figure 3A). Further, in our study, 44 couples had conceived through PGT and their embryonic karyotypes were analysed at the blastocyst stage prior to transfer. Our study confirmed that all karyotype results diagnosed by PGT were consistent with those detected using the NIPT method after embryo transfer.

**FIGURE 3:**
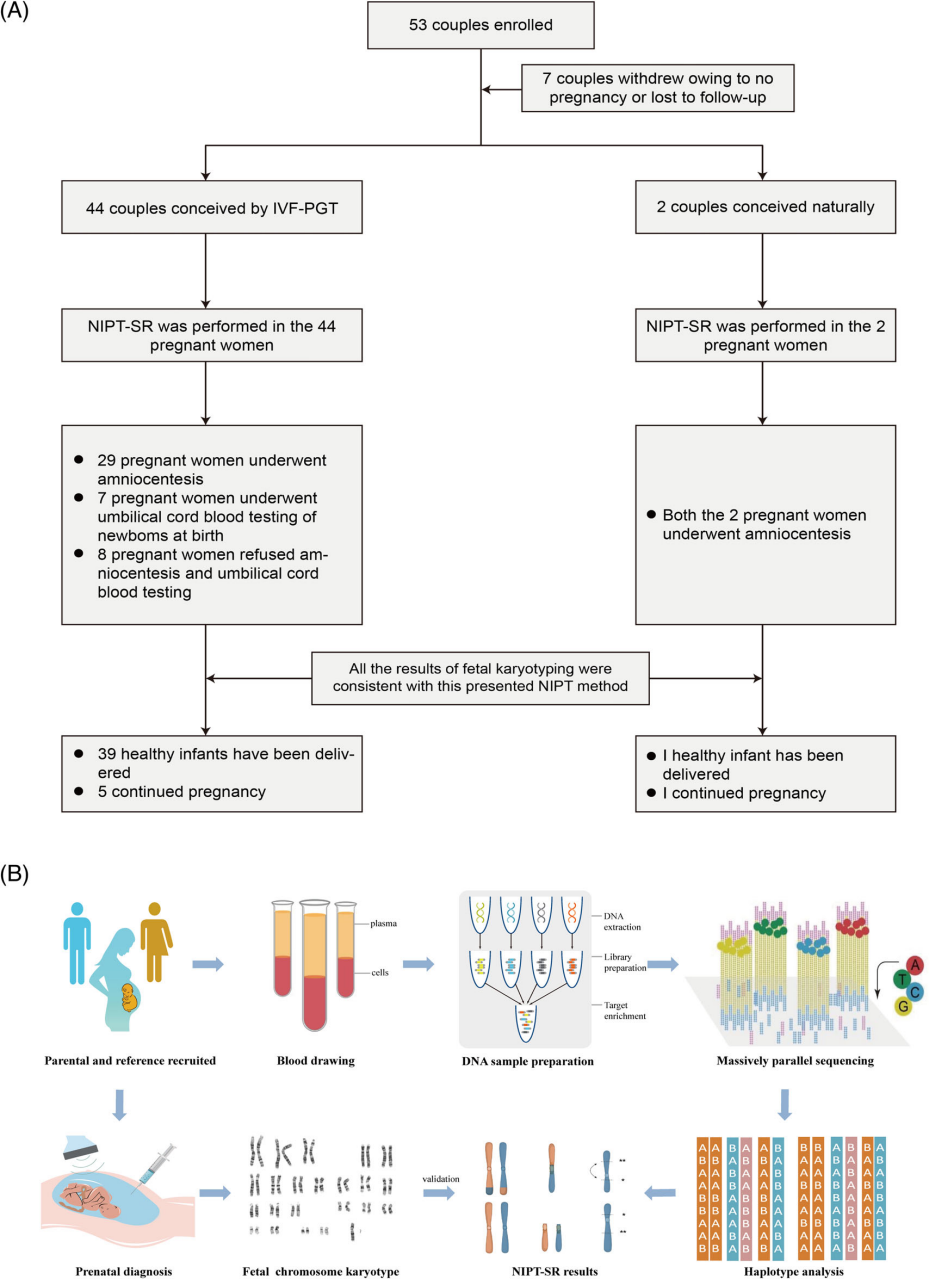
(A) The follow‐up outcome in the cohort. A total of 53 clinical samples were recruited initially in our centre, 7 patients withdrew this trial owing to no pregnancy or lost to follow‐up, and NIPT‐SR was performed in 46 couples finally. In these patients, 44 women got pregnant through IVF‐PGT and case‐22 and 29 were naturally pregnant. NIPT was conducted in all these 46 pregnant women, 29 pregnant women performed invasive prenatal amniocentesis at the second trimester and 7 performed umbilical cord blood testing of newborns at birth, the results of all foetal karyotypes were totally consistent with those detected by NIPT method. Till the revision of this manuscript, 40 healthy infants have been delivered and 6 continue the pregnancy. NIPT‐SR: Noninvasive prenatal testing for structural rearrangements. (B) The abstract figure of this study

The study strengths include its high accuracy and large sample size in the cohort, the rearranged chromosomes involve almost all 22 pairs of autosomes, proving that the noninvasive method is universal for different aspects of chromosomal rearrangements. To the best of our knowledge, this is the first report about a noninvasive approach to detect foetal balanced chromosomal rearrangements using maternal circulating cell‐free foetal DNA.

In conclusion, we develop a novel haplotype‐based NIPT method for couples with BCRs and demonstrated this approach is applicable and universal for detecting a wide spectrum of chromosomal rearrangements in foetus. The proposed new NIPT‐SR technology provides an option for these pregnant and will be an important supplement to the noninvasive prenatal diagnosis system for genetic diseases, reducing the possible miscarriage risk of IPD and facilitating the prevention of birth defects.

## CONFLICT OF INTEREST

The authors declare that they do not have any commercial or associative interest that represents a conflict of interest in connection with the work submitted.

## Supporting information

Supporting Information
