# Supplementary material for: A novel noninvasive prenatal testing method for chromosomal rearrangements using maternal circulating cell‐free foetal DNA
Source: Clin Transl Med. 2023 Jan 7;13(1):e1160. doi: 10.1002/ctm2.1160 (PMC9825107; doi:10.1002/ctm2.1160)
Supplement: Supplementary file 1 — Supporting Information [file CTM2-13-e1160-s001.docx]

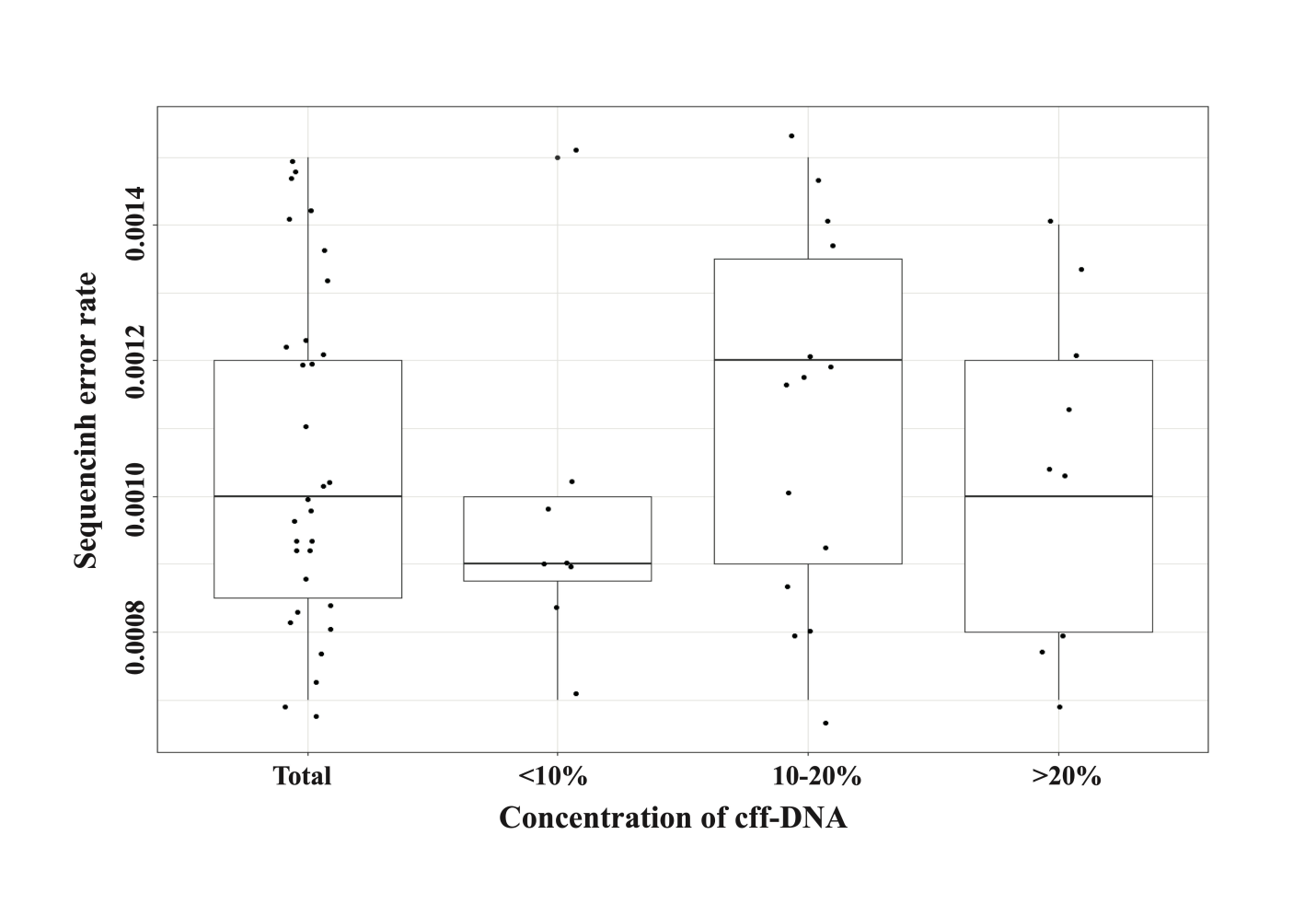


**Figure S1. The sequencing error rate among different cff-DNA concentration.** The cff-DNA concentration was divided into three groups, less than 10%, 10%-20% and more than 20%. The result indicated there was no statistically difference of sequencing error rate among these three groups.


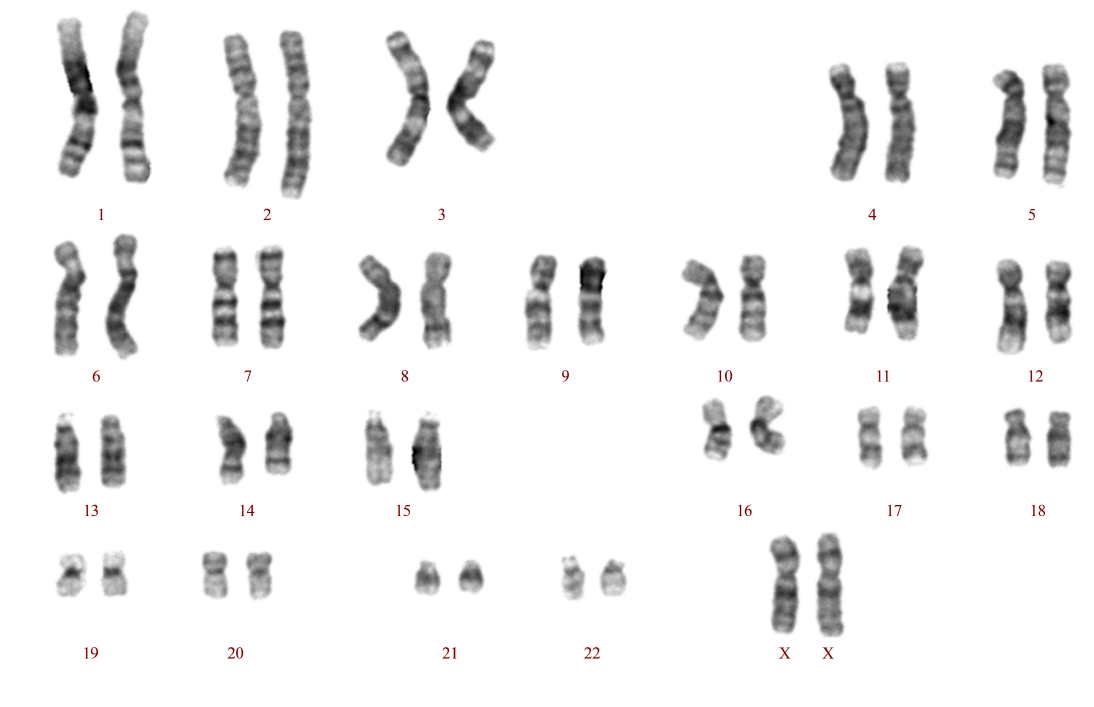


**Figure S2. The karyotype of fetal amniotic fluid cells in patient 6.** The karyotyping results of amniotic fluid cells showed the fetus was not a BCRs carrier, but has a normal karyotype.


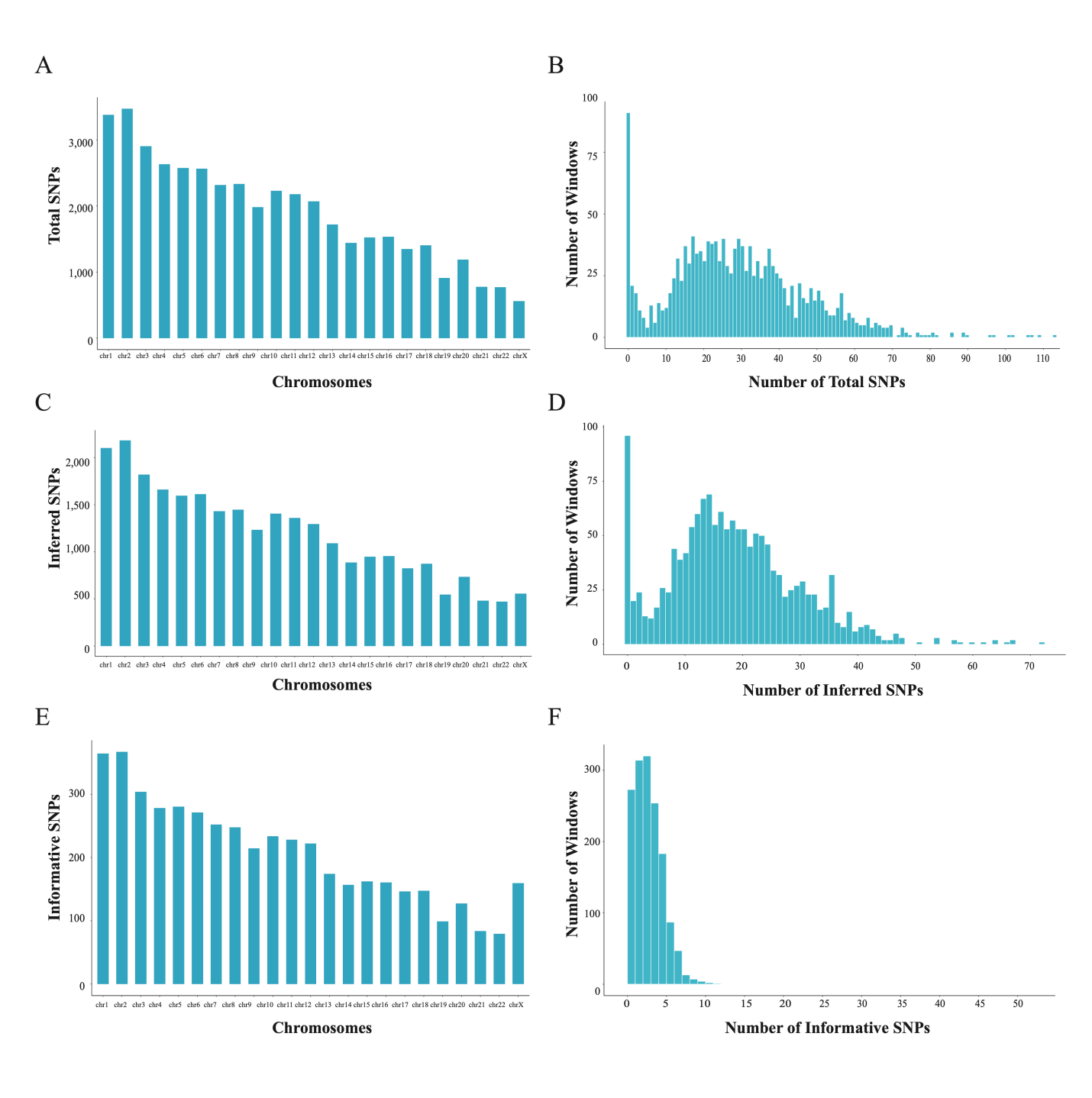


Figure S3. The distribution of SNPs in each chromosome and windows with different SNPs. (A) The distribution of total SNPs of our custom-designed capture panel in each chromosome. The horizontal axis represented the number of chromosome, and the vertical axis represented the number of total SNPs. (B) The distribution of total SNPs of the panel in windows. The horizontal axis represented the number of total SNPs, and the vertical axis represented the number of windows (2Mb region in genome). (C) The distribution of average inferred SNPs of our cases in each chromosome. The horizontal axis represented the number of chromosome, and the vertical axis represented the number of inferred SNPs. (D) The distribution of inferred SNPs in windows. The horizontal axis represented the number of inferred SNPs, and the vertical axis represented the number of windows. (E) The distribution of average informative SNPs of our cases in each chromosome. The horizontal axis represented the number of chromosome, and the vertical axis represented the number of informative SNPs. (F) The distribution of informative SNPs in windows. The horizontal axis represented the number of informative SNPs, and the vertical axis represented the number of windows.

| **Table S1. The karyotype results of patients’ parents** | | |
| --- | --- | --- |
| **Patient** | **Karyotype results of patients’ mother** | **Karyotype results of patients’ father** |
| Patient-1 | 46,XX,t(1;3)(q31;q23) |  |
| Patient-2 | 46,XX | 46,XY,t(1;5)(q32;q33) |
| Patient-3 | 46,XX | 46,XY,t(1;10)(q21;q26) |
| Patient-4 | 46,XX | 46,XY,t(1;12)(q21;p11) |
| Patient-5 | 46,XX | 46,XY,t(1;17)(p22;q23) |
| Patient-6 | 46,XX,t(1;20)(q25;p11.2) | 46,XY |
| Patient-7 | 46,XX | 46,XY,t(1;12)(q21;p11) |
| Patient-8 | 46,XX,t(2;8)(q33;q13) | 46,XY |
| Patient-9 | 46,XX,t(2;10)(q35;q22.1) | 46,XY |
| Patient-10 | 46,XX,t(2;10)(p21;p15) | 46,XY |
| Patient-11 | 46,XX,t(3;4)(p21;q35) | 46,XY |
| Patient-12 | 46,XX,t(3;7)(p11;q36) | 46,XY |
| Patient-13 | 46,XX | 46,XY,t(3;7)(p21;p15) |
| Patient-14 | 46,XX,t(3;6)(p10;p10) | 46,XY |
| Patient-15 | 46,XX | 46,XY,t(4;16)(q25;q13) |
| Patient-16 | 46,XX,t(4;18)(p14;p11) | 46,XY |
| Patient-17 | 46,XX,t(5,18)(q15,p11.2) | 46,XY |
| Patient-18 | 46,XX | 46,XY,t(5;13)(q15;p12) |
| Patient-19 | 46,XX,t(6;10)(q24;q23) | 46,XY |
| Patient-20 | 46,XX,t(9;12)(q31;q14) | 46,XY |
| Patient-21 | 46,XX,t(9;15)(p23;q25) | 46,XY |
| Patient-22 | 46,XX,t(9;17)(q34.2;p11.2) | 46,XY |
| Patient-23 | 46,XX | 46,XY,t(9,22)(q22,q11.2) |
| Patient-24 | 46,XX,t(10;17)(q26.3;q24) | 46,XY |
| Patient-25 | 46,XX | 46,XY,t(12;21)(p11.1;p11.1) |
| Patient-26 | 46,XX,t(13;14)(q32;q24) | 46,XY |
| Patient-27 | 46,XX,t(13;21)(q22;q22.1) | 46,XY |
| Patient-28 | 46,XX,t(13;22)(q21.2;q13.1) | 46,XY |
| Patient-31 | 46,XX | 45,XY,rob(13;14)(q10;q10) |
| Patient-32 | 45,XX,rob(13;14)(q10;q10) | 46,XY |
| Patient-33 | 45,XX,rob(13;14)(q10;q10) | 46,XY |
| Patient-34 | 45,XX,rob(13;14)(q10;q10) | 46,XY |
| Patient-35 | 45,XX,rob(13;14)(q10;q10) | 46,XY |
| Patient-36 | 46,XX | 45,XY,rob(13;14)(q10;q10) |
| Patient-37 | 46,XX | 45,XY,rob(13;14)(q10;q10) |
| Patient-38 | 46,XX | 45,XY,rob(14;15)(q10;q10) |
| Patient-39 | 46,XX | 45,XY,rob(14;21)(q10;q10) |
| Patient-40 | 46,XX | 45,XY,rob(14;21)(q10;q10) |
| Patient-41 | 46,XX | 45,XY,rob(14;21)(q10;q10) |
| Patient-42 | 45,XX,rob(14;21)(q10;q10) | 46,XY |
| Patient-43 | 46,XX | 45,XY,rob(14;21)(q10;q10) |
| Patient-44 | 46,XX | 46,XY,inv(7)(p21q21) |
| Patient-45 | 46,XX | 46,XY,inv(8)(p22q22) |
| Patient-46 | 46,XX,inv(18)(p11.2q21.1) | 46,XY |

| **Table S2.The accuracy of inferred SNP genotypes compared with direct fetal gDNA sequencing data** | | | | | |
| --- | --- | --- | --- | --- | --- |
| Patient | Maternal allele |  |  | Paternal allele |  |
|  | Total | Heterozygote |  | Total | Heterozygote |
| Chr1 | 99.04% | 98.47% |  | 100.00% | 100.00% |
| Chr2 | 99.74% | 99.92% |  | 99.73% | 99.86% |
| Chr3 | 99.14% | 99.14% |  | 99.85% | 99.85% |
| Chr4 | 99.14% | 99.60% |  | 99.79% | 100.00% |
| Chr5 | 97.56% | 98.89% |  | 99.89% | 99.85% |
| Chr6 | 98.32% | 98.67% |  | 99.95% | 100.00% |
| Chr7 | 98.21% | 98.84% |  | 99.67% | 99.61% |
| Chr8 | 93.51% | 95.33% |  | 100.00% | 100.00% |
| Chr9 | 95.77% | 95.90% |  | 99.53% | 99.43% |
| Chr10 | 97.83% | 98.07% |  | 100.00% | 100.00% |
| Chr11 | 99.19% | 99.45% |  | 99.85% | 99.76% |
| Chr12 | 99.36% | 99.20% |  | 99.79% | 100.00% |
| Chr13 | 98.54% | 98.05% |  | 99.73% | 99.56% |
| Chr14 | 99.47% | 99.79% |  | 100.00% | 100.00% |
| Chr15 | 99.37% | 99.22% |  | 99.84% | 99.75% |
| Chr16 | 97.67% | 97.64% |  | 99.73% | 100.00% |
| Chr17 | 96.67% | 95.99% |  | 99.92% | 100.00% |
| Chr18 | 99.79% | 100.00% |  | 99.84% | 99.83% |
| Chr19 | 93.89% | 91.95% |  | 99.79% | 99.80% |
| Chr20 | 94.45% | 95.48% |  | 99.90% | 100.00% |
| Chr21 | 93.52% | 94.81% |  | 100.00% | 100.00% |
| Chr22 | 98.86% | 99.02% |  | 100.00% | 100.00% |
| Total | 99.04% | 98.47% |  | 100.00% | 100.00% |
